# A new method for characterising shared space use networks using animal trapping data

**DOI:** 10.1007/s00265-022-03222-5

**Published:** 2022-08-26

**Authors:** Klara M. Wanelik, Damien R. Farine

**Affiliations:** 1 Department of Evolution, Ecology and Behaviour, Institute of Infection, Veterinary and Ecological Sciences, University of Liverpool, Liverpool, UK; 2 Department of Biology, University of Oxford, Oxford, UK; 3 Department of Evolutionary Biology and Environmental Studies, University of Zurich, Zurich, Switzerland; 4 Division of Ecology and Evolution, Research School of Biology, Australian National University, Canberra, ACT 2600 Australia; 5 Department of Collective Behaviour, Max Planck Institute of Animal Behavior, Konstanz, Germany

**Keywords:** Home range, Point-based observations, Social network, Space-sharing, Sparse observations, Trapping data

## Abstract

**Abstract:**

Studying the social behaviour of small or cryptic species often relies on constructing networks from sparse point-based observations of individuals (e.g. live trapping data). A common approach assumes that individuals that have been detected sequentially in the same trapping location will also be more likely to have come into indirect and/or direct contact. However, there is very little guidance on how much data are required for making robust networks from such data. In this study, we highlight that sequential trap sharing networks broadly capture shared space use (and, hence, the potential for contact) and that it may be more parsimonious to directly model shared space use. We first use empirical data to show that characteristics of how animals use space can help us to establish new ways to model the potential for individuals to come into contact. We then show that a method that explicitly models individuals’ home ranges and subsequent overlap in space among individuals (spatial overlap networks) requires fewer data for inferring observed networks that are more strongly correlated with the true shared space use network (relative to sequential trap sharing networks). Furthermore, we show that shared space use networks based on estimating spatial overlap are also more powerful for detecting biological effects. Finally, we discuss when it is appropriate to make inferences about social interactions from shared space use. Our study confirms the potential for using sparse trapping data from cryptic species to address a range of important questions in ecology and evolution.

**Significance statement:**

Characterising animal social networks requires repeated (co-)observations of individuals. Collecting sufficient data to characterise the connections among individuals represents a major challenge when studying cryptic organisms—such as small rodents. This study draws from existing spatial mark-recapture data to inspire an approach that constructs networks by estimating space use overlap (representing the potential for contact). We then use simulations to demonstrate that the method provides consistently higher correlations between inferred (or observed) networks and the true underlying network compared to current approaches and requires fewer observations to reach higher correlations. We further demonstrate that these improvements translate to greater network accuracy and to more power for statistical hypothesis testing.

**Supplementary Information:**

The online version contains supplementary material available at 10.1007/s00265-022-03222-5.

## Introduction

Social networks are central to addressing many of the key questions in ecology and evolution (Cantor et al. 2021). However, network construction remains a major challenge in many systems because large numbers of observations are needed to construct meaningful networks (Whitehead 2008a; Farine and Whitehead 2015). Recent technological improvements for collecting proximity, contact, or interaction data allow much more detailed networks to be constructed by improving the temporal resolution at which the data are collected (Douglas et al. 2006; Rutz et al. 2012; Ryder et al. 2012; Berkvens et al. 2019). However, for many smaller or more cryptic species, where observation remains difficult, many studies still rely on collecting data by trapping individuals and inferring indirect and/or direct contacts from observations of different individuals occurring in the same trap at different times (i.e. sequential trap sharing events; e.g. Perkins et al. 2008; Porphyre et al. 2008; Grear et al. 2009; Grear et al. 2013; VanderWaal et al. 2013; Davis et al. 2014). To date, no study has quantified whether the sparse observations typical of such studies allow us to construct robust social networks (an issue highlighted in relation to disease transmission; Tompkins et al. 2011; White et al. 2017) and, therefore, whether we can extract meaningful biological relationships from these networks.

Sequential trap sharing networks can be constructed using a range of different data collection methods that record the location of individuals at particular times through trapping events, most commonly live-capture traps and camera traps, but also increasingly using RFID detections (Sabol et al. 2018). These methods are characterised by having the capability to observe multiple individuals in the same location(s) over time. Individuals that are then observed (trapped) at the same location are considered to be connected, with binary edges (there or not) between them, or, if constructing a weighted network, with the number of detections or number of locations at which both individuals were recorded defining the strength of their connection. However, trapping typically only detects single individuals at any one time, hence why we call these ‘sequential trap sharing events’. When using such data to construct networks (herein sequential trap sharing networks), studies such as those listed above assume that two individuals that are detected in the same location are more likely to have had some form of contact (some studies, such as VanderWaal et al. 2013, use such an approach to solely model the potential for indirect contact). However, here we argue that sequential trap sharing events, and resulting sequential trap sharing networks, are unlikely to provide accurate information about direct or indirect contacts among individuals.

The ability to construct robust contact networks from sequential trap sharing events will heavily depend on how well detecting two individuals in the same trap, sequentially, generalises to the patterns of space-sharing between the two individuals away from trapping locations. On the one hand, observing two individuals in the same trapping location provides some certainty that they have come into indirect contact with each other (at least within some informative timeframe). Given sufficient amounts of data, it is then plausible to assume that the tendency to be detected in the same location will also correlate with the tendency to have direct contact. On the other hand, observations of animals at the same location could easily over-estimate the chance of direct and/or indirect contact between individuals. Take, for example, a trap that sits at a shared boundary between two individuals’ home ranges, detecting both individuals. If these are also detected once each at two additional traps in each of their respective home ranges, then we would infer an edge weight of 0.2, whereas in reality, the actual area of overlap is negligible. Thus, sequential trap sharing networks may be prone to error at low trapping rates. Such insights were previously also reported for telemetry-based networks (Gilbertson et al. 2021).

Constructing a sequential trap sharing network then tacitly invokes the assumption that repeatedly observing two individuals in the same trap (at different times) informs us about their broader likelihood of sharing space away from trapping locations. When using sparse datasets, due to individuals being rarely trapped, this inference is substantially limited by stochasticity because they can only capture a tiny proportion of all true space-sharing events. These limitations therefore raise the question of whether these data may be better modelled under a different set of logic and assumptions.

One potential solution is to use trapping data to explicitly model shared space use. Spatial overlap networks take a different order of inference to sequential trap sharing networks. First, observations (e.g. trapping data) are used to characterise individual-level space use. Only then are these space use data linked to those of other individuals in order to infer the amount of spatial overlap between individuals, where edge weights range from 0 if two individuals have no spatial overlap to 1 if they completely overlap. This assumes that the more they overlap, the more they are likely to have some amount of contact. This could have several advantages. First, given that methods for estimating space use can require as few as three observations per individual (to create a polygon), it is possible that first estimating space use and then estimating spatial overlap could be a relatively powerful approach when data are limited. Second, spatial overlap networks may rely less on high trapping rates as, for example, trapping two individuals with overlapping ranges in interspersed traps (but never in the same trap) can still inform spatial overlap networks, but would incorrectly suggest no potential for (direct or indirect) contact when using a sequential trap sharing network. Despite these potential benefits, to date, there has been no direct quantification of the robustness of either sequential trap sharing or spatial overlap networks and how these might perform under different sampling regimes.

It has been suggested that the data-intensive nature of networks may act as a barrier to the more widespread use of networks in the fields of ecology and evolution, with wildlife systems often being data limited (Craft and Caillaud 2011). Recent investigations into networks based on co-occurrence data (Farine and Strandburg-Peshkin 2015; Hart et al. 2022) and direct observation methods (Davis et al. 2018) have highlighted how data-hungry networks are. Constructing a meaningful network requires sufficient observations to accurately estimate each of the many relationships (both present and absent) that connect all individuals in a population (specifically: $\frac{n(n-1)}{2}$ edges in an undirected network). Thus, the sampling intensity needed to maintain a minimum number of observations per individuals and, critically, the co-observations of pairs of individuals (dyads) grows quadratically with the number of individuals represented in a given network. By contrast, quantifying individual space use would result in a linear relationship between population size and sampling effort.

Previous studies quantifying the data required to construct meaningful social networks from co-occurrence data have suggested that a good rule of thumb is that an average of 15 opportunities to co-observe all pairs of individuals are needed (i.e. of potential associations or interactions, which could be both individuals together or just one individual in the absence of the other; Farine and Strandburg-Peshkin 2015; Davis et al. 2018). Importantly, more observations are required for accurately defining network structure when the differences in the relationship among dyads are more uniform through a population (Whitehead 2008a, b; Hart et al. 2022), such as we expect in less social species. However, these estimates of effort may not translate well to quantifying shared space use networks, as space use is a property that can be characterised at the level of the individual.

Addressing the question of how much data need to be collected is also crucially important because many studies that have constructed sequential trap sharing networks do not report the mean number of observations per individual (Webber and Vander Wal 2019). Furthermore, the majority of those that do have been based on relatively few observations per individual. In one of the better examples, VanderWaal et al. (2013) had a mean of 11 trapping events per individual, meaning that they would have, at most, a mean of 22 potential observations from which to characterise dyadic edge weights in their network (observing two individuals apart 11 times each would give the denominator of an association index—e.g. the proportion of time individuals were associated—of 22, see Hoppitt and Farine 2018). The sparser the observations (e.g. trapping events), the less likely it is that detecting two individuals at the same trap on different days is likely to capture information about the real contacts between those individuals. Understanding how the number of trapping events relates to the robustness of network estimates remains a major gap in knowledge.

The choice of which data to include when constructing networks from sequential observations of individuals in space (e.g. trapping data) can also have an impact on how meaningful the resulting network is. Many studies have used sequential trap sharing networks to study the potential for indirect transmission events. These have typically defined a temporal threshold within which the observation of two individuals in the same place must occur for these observations to be counted as a connection. The choice of threshold is directly inspired by biology, commonly the lifetime of a disease vector or pathogen in studies using networks to characterise indirect disease transmission. For example, Porphyre et al. (2008) used 28 days (maximum survival of *Mycobacterium bovis* in the environment) and Perkins et al. (2008) used 14 days (the time needed for an infective L3 larval stage of *Heligmosomoides polygyrus* to develop from the eggs of an infected host). However, such temporal definitions can be at odds with the definition and biological motivation behind applying a network approach.

For studies that rarely observe individuals, but where individuals have relatively stable ranging areas, trapping data are most powerful when used to define the potential for individuals to co-occur (and possibly encounter one another) anywhere within their respective home ranges, rather than whether they actually did co-occur (and possibly encounter one another) at the particular location where the trap was set. Some studies can regularly (and almost synchronously) observe or recapture individuals (such as Smith et al. 2018 who used PIT-tag readers at the entrance of burrows), and are therefore able to directly relate observed space sharing events (e.g. within a burrow on a given visit) to indirect contacts, or even direct contacts if the temporal gap between detections is very short. This is because observations of individuals, and subsequently space sharing events, are occurring at the same spatial and temporal scales as disease transmission events or social behaviours that form part of the study of interest (see Farine 2018 for further discussion). Restricting the temporal scale for defining connections also reduces the data available from which the strength of connections between individuals can be estimated, which is counter-productive when animals are relatively sedentary. For example, a study that traps individuals once every few months, and discards a detection between two individuals in the same trap 15 days apart because of a maximum 14-day transmission period, would be putting too much certainty on the time-gap in detections relative to the certainty they have in terms of detecting individuals in the first place. Using observations spaced more widely apart in time forms connections between individuals that describe the system more generally as opposed to attempting to precisely quantify the actual connections among individuals (although these should correlate given sufficient observations, e.g. Sabol et al. 2018).

In this study, we conduct a quantitative evaluation of the robustness of sequential trap sharing and spatial overlap networks to different sampling regimes. We first use empirical data to highlight that characteristics of how animals use space can help us to establish new ways to model the potential for individuals to co-occur (and potentially encounter one another). We then describe a new method for characterising shared space use network that more generally estimates home range overlap. We show that using this method can generate a network that generally (1) is more strongly correlated with the true shared space use network, (2) is a more accurate representation of the true space sharing network and, therefore, (3) has greater power to detect biological effects present in the true shared space network, relative to sequential trap sharing networks. Importantly, the spatial overlap method requires many fewer observations than using sequential trap sharing networks to reconstruct meaningful shared space use networks and many fewer observations than what has been suggested in the more general guidelines for social networks (e.g. 15 potential observations per dyad; see above). We also confirm that the approach is relatively robust to the underlying home range characteristics of animals. Finally, we discuss the topic of inference from shared space use networks, and how appropriate it is to link network data with biological processes.

## Materials and methods

Our study consists of three core components. First, we use a large empirical dataset to highlight characteristics of how animals use space, specifically that they have a core and a periphery to their home range. Second, we estimate the ability for data on sequential trap sharing events by individuals to generate shared space use networks that are robust to different sampling regimes using simulated data. Third, we describe a new method, inspired by the core-peripheral nature of animal home ranges, for defining network edges. We use the same simulated data to show that this spatial overlap method generates observed shared space use networks that are, for a given number of captures per individual, more strongly correlated with the true shared space use network, are a more accurate representation of the true shared space use network, and are more powerful at detecting biological effects present in the true shared space use networks relative to networks generated directly from the observed sequential trap sharing events. We further demonstrate that spatial overlap networks can be constructed using different methods for estimating individual space use with resulting network varying in their performance and that our findings are robust to our modelling assumptions. All simulations were run in R version 3.3.1 (R Core Team 2016) using the packages vegan version 2.4–3 (Oksanen et al. 2017) and sna version 2.4 (Butts 2016).

### Modelling home ranges in an empirical dataset

There is a large body of literature on how best to model animal space use, and it is widely accepted that many animals have a core and a periphery to their home range (Hayne 1949, 1950; Calhoun and Casby 1958; Jennrich and Turner 1969; Schoener 1981; Swihart and Slade 1989; Spencer et al. 1990; Slade and Russell 1998; Zamora and Moreno-Amich 2002; Klein and Cameron 2012). We test whether this holds true in a large-scale empirical dataset for a population of field voles (*Microtus agrestis*), and in so doing, the utility of this simple representation of home range for modelling the space-use behaviour of large numbers of animals.

We use part of a dataset from a study of *M. agrestis* in Kielder Forest, UK (55°13′ N, 2°33′ W) that involved capturing individuals using live-trapping methods. Access to the study site was provided by the Forestry Commission. Full details are given in Jackson et al. (2014). The site was monitored across 2 years (2009–2010) by monthly trapping sessions between February and November and contained a live-trapping grid (0.375 ha) of 150 (10 × 15) regularly spaced traps (at 3–5 m intervals) placed in optimal habitat. Animals were marked with passive radio frequency transponders (AVID plc, East Sussex, UK) and monitored over time, thus providing sequences of capture and recaptures. This dataset is comprised of 347 individuals and 678 trapping events. Because we used a published dataset, we were blind to any treatments undertaken as part of the original study.

*M. agrestis* is a polygynous species, with strictly territorial males. Home ranges of *M. agrestis* vary across different locations, habitats and across different times of the year. A review of nine studies, all conducted in later summer, but across a range of locations and habitats and using a range of different home range estimation methods, found that female home ranges varied in size from 30 to 900 m^2^ while males home ranges varied in size from 200 to 1500 m^2^ (Borowski 2003). Large males also have the largest home ranges (Borowski 2003). In our own study population, there is evidence for differences in the degree to which large males, small males and females are discouraged by distance (Davis et al. 2014). We estimate home range parameters for females and large males (mean weight ≥ 25 g) as an example, which we go on to use in our simulations (see the ‘Simulation procedure’ section).

### Simulation procedure

**Fig. 1:**
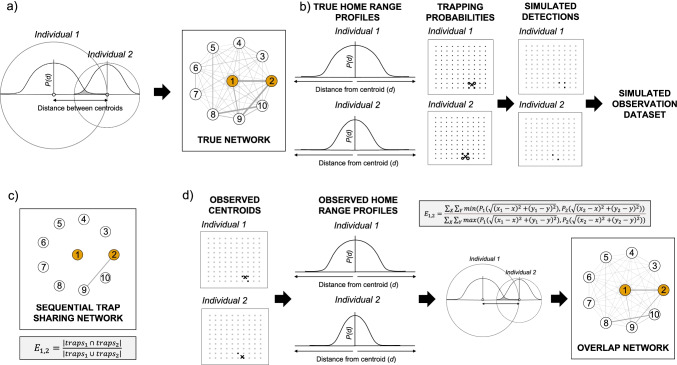
Schematic showing the simulation process: **a** Simulation of the true shared space use network with edge weights between two individuals (e.g. individual 1 and individual 2) equal to the overlap between their respective home ranges (here depicted along a one-dimensional slice). Each home range modelled using a negative sigmoidal curve with class-specific parameters (*a* and *b*; Eq. 1) that captures the decreasing probability of observing individuals as the distance away from the centroid increases, and the overlap between the two-dimensional surfaces produced by the negative sigmoidal curves being calculated using Eq. 2. **b** Generating a simulated observation dataset by calculating the probability for a given individual to be observed in a given trap based on its home range profile (crosses represent centroids; circles represent trapping probabilities; the bigger the circle, the greater the probability of detecting an individual at a trap), then simulating observations by drawing from a binomial distribution {0,1} with the probability of getting a 1 for a given individual in a given trap defined by this trapping probability. **c** Generating a sequential trap sharing network, where nodes represent individuals and where edge weights are calculated using Eq. 3. **d** Generating an overlap network, where nodes represent individuals, and where edge weights represent the overlap between two individuals’ observed home range profiles. First, the observed centroid was calculated for each individual. Then, we modelled class-specific home ranges using a negative sigmoidal curve (using a GLM regression). Third, we used Eq. 2 to calculate home range overlaps as an estimate of shared space use (i.e. the edge weights), as in **a** but with the observed home range profiles and centroid values

In brief, our study used the following procedure (see Fig. 1):

1. We simulated a set of 100 individuals with home ranges defined by a centroid and characterized by a negative sigmoidal curve that highlights the declining probability *P* of an individual to be detected at an increasing distance (*d*) away from the centroid of its home range:
  1 $$P(d)= \frac{1}{1+{e}^{-a-bd}}$$
  where *a* describes the overall size of the home range, *b* describes the steepness of the edge of the home range and $d$ is the logarithmic distance from the centroid. Our choice of negative sigmoidal curve was inspired by the core-peripheral nature of animal home ranges, and a large-scale empirical dataset for a population of field voles (*M. agrestis*), but we validate that our results are robust even when home ranges are defined using a uniform distribution. We defined the true shared space use network as the amount of overlap in the home range profiles across all combinations of individuals (see detailed methods below).
2. We randomly placed simulated individuals in a spatial area containing *T* traps laid out in a stratified grid. We then simulated observation datasets that contained detections of individuals at traps, where the detection probability for a given individual in a given trap was determined by the position of the trap relative to the home range profile of the individual defined in Eq. 1 (higher closer to the centroid, lower further away from the centroid) or using a uniform distribution centered on the individual centroids.
3. From the simulated observation datasets, we constructed a sequential trap sharing network.
4. Finally, we applied a novel method to construct a spatial overlap network, based on estimating individual home ranges and estimating home range overlap among individuals.

Below we describe steps 1–4 in more detail:

1. Simulating true networks
  We first drew *N* sets of *x* and *y* coordinates from a uniform distribution, where the boundaries of the distribution correspond to the edges of our study area (in our case, from 0 to 10 in each dimension). For each individual, we also randomly allocated a sex (male or female) and drew home range parameters (*a* and *b* in Eq. 1) based on the sex, giving males a larger home range than females. Home range parameters for males and females were based on the empirical data (see ‘Modelling home ranges in an empirical dataset’ section), with added noise drawn from a normal distribution with standard deviation equal to 0.05 times the home range parameter in question (*a* or *b*) to simulate individual-level variation in home range profile.
  For each simulation, we generated a true shared space use network, with edge weights representing the amount of overlap in the home range between each pair of individuals. This was done numerically by overlaying the two individuals’ 2D home range profiles and calculating the area under the two surfaces (Fig. 1a). Specifically, we predicted the probability of detecting each individual in a grid overlapping both their home ranges, using Eq. (1), and calculated the overlap (the edge weight between individuals 1 and 2, ${E}_{\mathrm{1,2}}$) by dividing the sum of the lowest values at each point on the grid (*x, y*) by the sum of the largest values at each point, according to the following equation:
  2 $${E}_{\mathrm{1,2}}=\frac{{\sum }_{X}{\sum }_{Y}\mathrm{min}({P}_{1}(\sqrt{{\left({x}_{1}-x\right)}^{2}{+\left({y}_{1}-y\right)}^{2}}),{P}_{2}(\sqrt{{\left({x}_{2}-x\right)}^{2}{+\left({y}_{2}-y\right)}^{2}})) }{{\sum }_{X}{\sum }_{Y}\mathrm{max}({P}_{1}(\sqrt{{\left({x}_{1}-x\right)}^{2}{+\left({y}_{1}-y\right)}^{2}}),{P}_{2}(\sqrt{{\left({x}_{2}-x\right)}^{2}{+\left({y}_{2}-y\right)}^{2}}))}$$
  where ${P}_{n}\left(\sqrt{{\left({x}_{n}-x\right)}^{2}{+\left({y}_{n}-y\right)}^{2}}\right)$ is the probability of observing individual $n$, with a home range centred at (${x}_{n}, {y}_{n}$), at location (*x, y*) from Eq. 1. $X$ and $Y$ represent the set of grid coordinates that encompass the home ranges of both individuals or the range of coordinates covering the entire study area, with the spacing between grid points being substantially smaller than the home ranges (e.g. every 0.1 m).
  To confirm that our results are not dependent on the definition of the true network, we also repeat our simulations by generating square home ranges with a uniform probability across their range. We do so by setting female home ranges to a 2.5 × 2.5 square, and male home ranges to a 3.5 × 3.5 square, roughly approximating differences in the observed empirical home ranges (results presented in Supplementary information).
2. Simulating observations of individuals in traps
  We first calculated the probability for a given individual to be observed in a given trap. We defined this probability based on the distance of the trap to the centre of the individual’s home range using Eq. 1 (or giving a uniform probability to each trap within the home range when using uniform home ranges). We repeatedly did this for all combinations of individuals and traps (‘trapping probability’ in Fig. 1b). We then used these probabilities to simulate observations by drawing from a binomial distribution {0,1} (‘simulated detections’ in Fig. 1b). We incremented the number of draws from this sampling process to generate more observations. Because draws resulted in variable numbers of observations, we then calculate the mean number of observations per individual, allowing us to make our results more easily interpretable.
3. Generating sequential trap sharing networks
  Each simulated dataset contained the number of detections of each individual in each trap. We generated a sequential trap sharing network for each simulated dataset with the edge weight between individual 1 and individual 2 (${E}_{\mathrm{1,2}}$) defined as follows:
  3 $${E}_{\mathrm{1,2}}=\frac{\left|{\mathrm{traps}}_{1 }\cap { \mathrm{traps}}_{2}\right|}{\left|{\mathrm{traps}}_{1 }\cup { \mathrm{traps}}_{2}\right|}$$
  where ${\mathrm{traps}}_{1 }\cap { \mathrm{traps}}_{2}$ is the set of traps in which both individuals were detected, and ${\mathrm{traps}}_{1 }\cup { \mathrm{traps}}_{2}$ is the set of traps in which either or both individuals were detected (Fig. 1c).
4. Generating networks based on overlapping home ranges
  We then applied a novel method for generating shared space use networks based on first estimating a population’s home range profile(s) from a simulated observation dataset and then calculating the overlap in the observed home range profiles of each pair of individuals based on the distance between their observed centroids. Our method operates as follows. First, we calculate each individual’s observed centroid by taking the mean of all of the detected locations. Second, we calculate the distance between this centroid and all of the traps where it could have been captured. Third, we calculate the observed home range profile for individuals, which (for a representative, sparse dataset) we achieve by fitting a negative sigmoidal curve (Eq. 1; fitted using a Bernoulli GLM, with 0 indicating an individual was not detected at a particular trap, and 1 indicating an individual was detected at a particular trap) for males and females separately, thereby generating a relationship representing the average home range profile for each sex (see discussion for justification for this strategy of combining individuals, as well as alternative strategies). Fourth, we use the observed profiles calculated for each individual to estimate overlap in space use between each pair of individuals using Eq. 2 (Fig. 1d).
  We also tested whether spatial overlap networks are robust to different implementations by calculating home ranges using minimum convex polygons (MCPs) and estimating pairwise overlaps between individuals’ polygons (these results are presented in the Supplementary information).

### Estimating the robustness of shared space use networks to different sampling regimes

The ultimate aim of any network we construct is to be able to reliably test a hypothesis of interest. To test the performance of our novel spatial overlap network method against traditional sequential trap sharing networks, we generated 1000 true networks (Fig. 1a). For each true network, we produced simulated observation datasets that varied in sampling intensity (number of draws from a binomial distribution {0,1} given a probability of observing an individual in a trap; Fig. 1b). We designed this such that the sampling intensity corresponded to a mean number of observations per individual ranging between 1 and 30 (regardless of trapping grid density), thus capturing the spectrum of what has been reported in the literature. For each simulated observation dataset, we generated a sequential trap sharing network and a network using the spatial overlap approach by reconstructing separate negative sigmoidal curves (or home range profiles) for large males and females (Fig. 1c).

We assessed the performance of each of these observed shared space use networks for three metrics. First, we calculated the correlation between the edge weights in the observed network and the edge weights in the true network using a Mantel test. The correlation provides a measure of relative position of each edge, such that when the correlation is 1, the position of each edge from the observed network is the same as the positions from the true network, irrespective of any changes in scale. Second, we calculated a measure of accuracy by taking the mean of the absolute differences between the observed and true network edge weights. The accuracy provides a measure of whether the estimated edges in the observed network are on the same scale as those in the true network. Third, we calculated a measure of power by finding the proportion of observed networks in which we could detect a significant biological effect—here the difference in mean degree (sum of edge weights) between large males and females (large males were given a larger home range than females, see point 1 of the simulation procedure)—that is present in the true network (and estimated false positives by re-running the simulations without any difference between large males and females). We estimated significance for each simulated observation dataset by comparing the observed difference in mean degree between large males and females to the distribution of differences in 100 permuted networks. We used node permutations, which involved randomising the assignment of sex to the identities of each individual. We deemed the effect from an observed network to be significant if fewer than three of the randomised networks generated a difference that was larger than the observed one (two-tailed test at *p* = 0.05; see Farine 2017).

#### Variants

We repeated the procedure described above for true networks with varying effect sizes for the difference in mean degree (sum of edge weights) between large males and females by varying the *b* parameter, resulting in the following: (a) an effect size half that in our empirical dataset, (b) an effect size equal to that in our empirical dataset, (c) an effect size twice that in our empirical dataset (see the ‘Results’ section). We also repeated the procedure using trapping grids of differing densities: (a) a 10 × 10 grid and (b) a 19 × 19 grid within the same area.

## Results

### Modelling home ranges in an empirical dataset

**Fig. 2:**
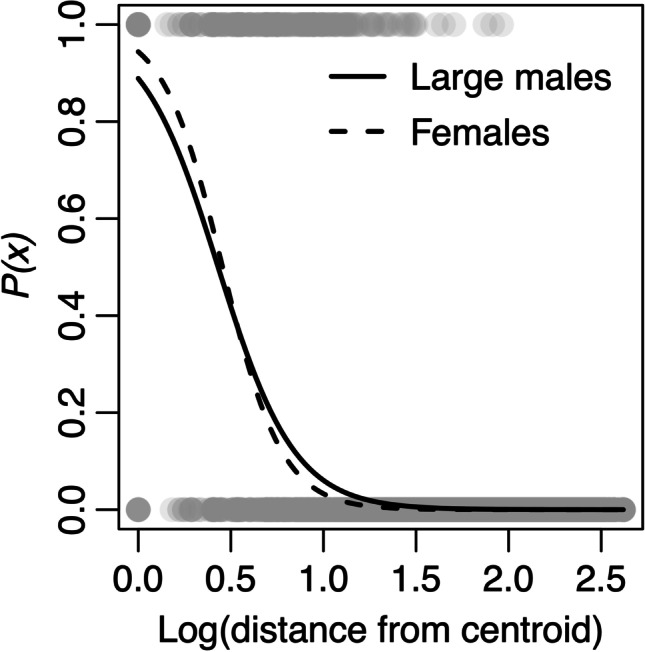
Class-specific negative sigmoidal curves for *M. agrestis* describing the change in probability of detection with increasing distance from the centre of an individual’s home range. Line shows the fitted home range profile for large males and females. Points show the raw data (whether, 1, or not, 0, an individual was detected at a location). Distances are measured in trapping grid cells (1 grid cell = 3–5 m)

Consistent with space use theory, we found evidence for a declining probability of an individual field vole to use space further away from the centre of its home range. Furthermore, we characterised this empirical relationship, between probability of detection and distance from centroid, using a negative sigmoidal curve (Eq. 1; fitted using a Bernoulli GLM, with 0 indicating an individual was not detected at a particular trap, and 1 indicating an individual was detected at a particular trap). We found evidence for large males having a larger home range (*a* = 2.08, *b* =  − 4.82) than females (*a* = 2.83, *b* =  − 6.21; interaction term between sex and distance from centroid: *p* < 0.001; Fig. 2), resulting in a difference in mean degree of 1.8 between these two classes. We used these class-specific curves, and the resulting difference in mean degree, as the primary method (presented in the main text) to generate true shared space use networks in our simulations.

### Performance of simulated observed networks with varying numbers of captures per individual

**Fig. 3:**
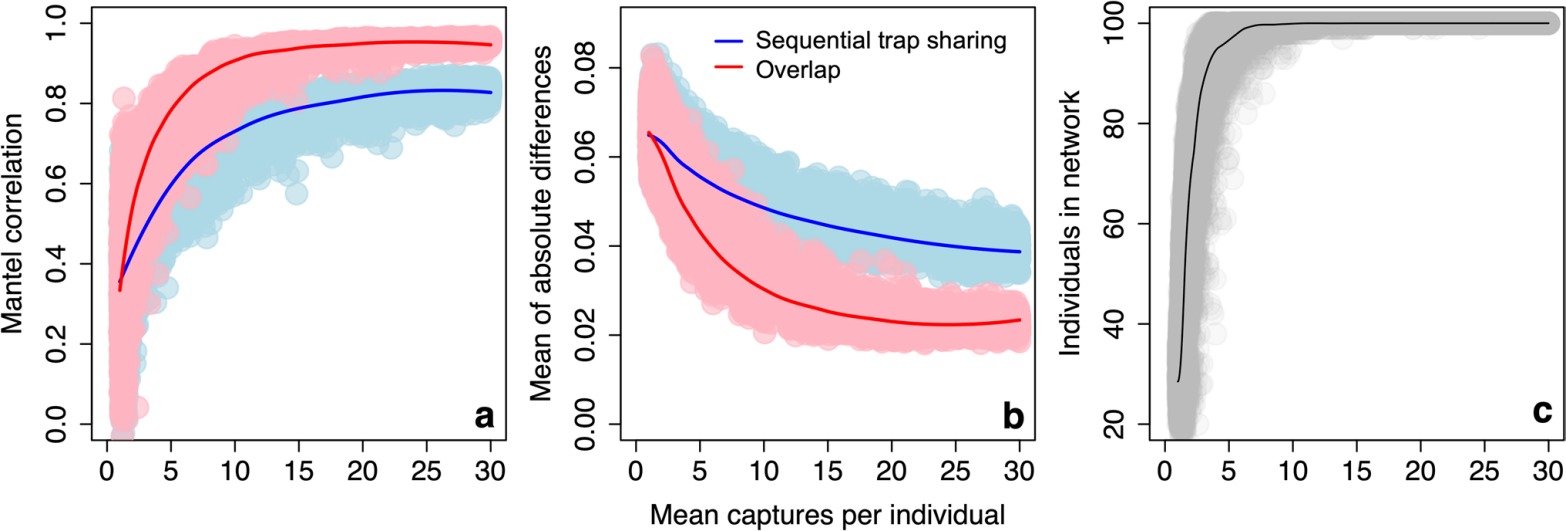
Performance of observed networks with varying numbers of captures per individual on a 10 × 10 trapping grid, as measured by **a** correlation, Mantel correlation between edge weights in observed and true networks; **b** accuracy, mean of absolute differences in edge weights between observed and true networks (lower values = more accurate networks); and **c** number of individuals in observed networks. Individual data points are plotted and a LOESS smoother added to aid visual interpretation. Panel **c** refers to the data in the simulated observation dataset, which is identical for both methods

The number of individuals detected at least once at a trap increases with the number of captures per individual, starting from a mean of 31.3 individuals (out of a total population of 100 individuals) present at a mean of 1.3 captures per individual, and reaching a mean of 100.0 individuals (i.e. the whole population) present at approximately 10 captures per individual (Fig. 3c).

#### Correlation

As the mean captures per individual increases, the sequential trap sharing network becomes more strongly correlated with the true network. At a mean of 1.9 captures per individual, the Mantel correlation coefficient between the sequential trap sharing network and the true network is 0.4. The correlation coefficient plateaus from a mean of approximately 20 captures per individual, reaching a maximum of 0.8 at a mean of 28.7 captures per individual. The spatial overlap network shows broadly the same pattern, but is, for a given number of captures per individual, typically more strongly correlated with the true network than the sequential trap sharing network. At a mean of 1.9 captures per individual, the correlation coefficient between the spatial overlap network and the true network is 0.5. The correlation coefficient also plateaus earlier, from a mean of approximately 10 captures per individual, and reaches a higher maximum of 1.0 (Fig. 3a).

#### Accuracy

As the mean number of captures per individual increases, the sequential trap sharing network becomes more accurate. At a mean of 1.9 captures per individual, the mean of the absolute differences in edge weights between the true network and sequential trap sharing networks is 6.4 × 10^−2^, which reaches a minimum of 3.9 × 10^−2^ at a mean of 28.7 captures per individual. The spatial overlap network shows broadly the same pattern, but is more accurate for a given number of captures per individual. For example, at a mean of 1.9 captures per individual, the absolute difference in edge weights to the true network is 6.1 × 10^−2^. The mean of differences for the spatial overlap network also reaches a lower minimum of 2.3 × 10^−2^ (Fig. 3b).

#### Power

**Fig. 4:**
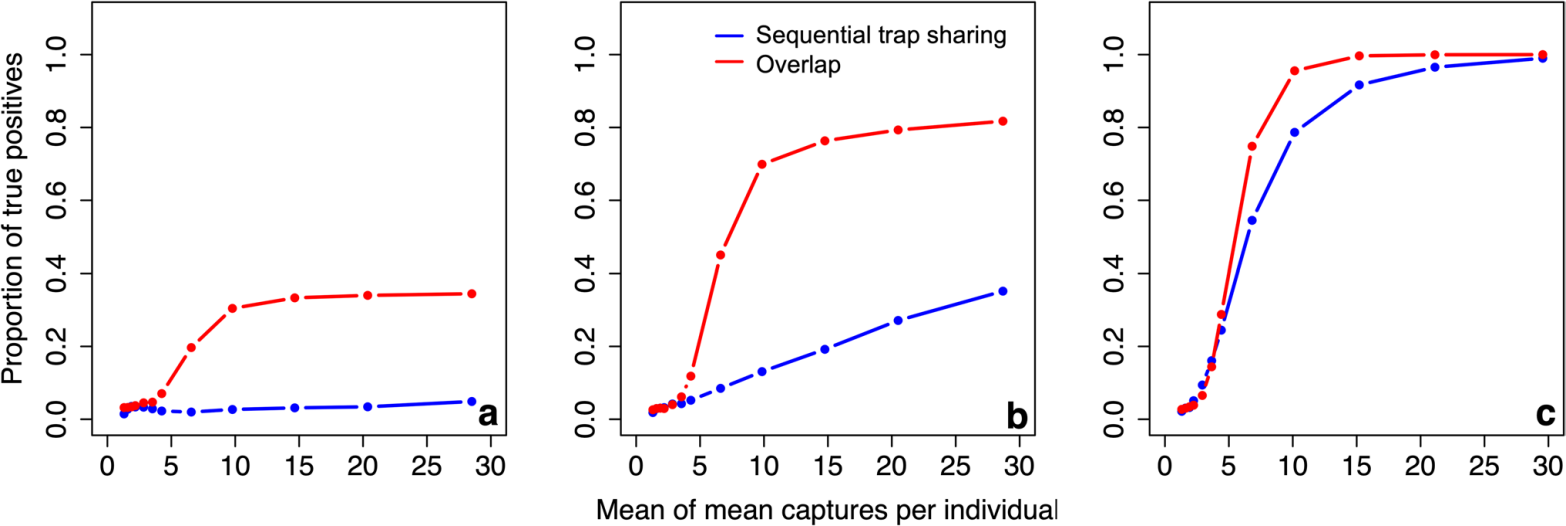
Performance of observed networks with varying numbers of captures per individual on a 10 × 10 trapping grid, as measured by the power of observed networks to detect a biological effect present in the true network. Proportion of true positives shown on *y*-axis, and mean of mean captures per individual shown on *x*-axis. Repeated for true networks with varying effect sizes: **a** an effect size half that in our empirical dataset, **b** an effect size equal to that in our empirical dataset, **c** an effect size twice that in our empirical dataset

As the mean number of captures per individual increases, the ability to detect a true biological relationship (i.e. the power) of the sequential trap sharing network also increases. For example, there is nearly double the chance of detecting a true positive at a mean of 4.3 captures (5.2%) compared to 1.9 captures (3.1%). However, the power remains low for small effect sizes even after large numbers of captures. The spatial overlap network shows broadly the same pattern, but has consistently greater power to detect an effect for a given number of captures per individual, above a mean of approximately 3 captures per individual. For example, at 4.3 captures per individual there is more than double the chance of detecting a true positive in the spatial overlap network (11.8%) compared to the sequential trap sharing network (5.2%). The power of the sequential trap sharing network increases continuously and reaches a maximum power of 35.2% at a mean of 28.7 captures per individual. The power of the spatial overlap network plateaus at a mean of approximately 10 captures per individual, reaching a much higher maximum of 81.7% (Fig. 4a). Below 3 captures per individual, the sequential trap sharing and spatial overlap networks have similar power to detect an effect.

Below 5 captures per individual, the sequential trap sharing and spatial overlap networks have a similar rate of false positives. At a mean of 9.7 captures per individual, the false positive rate of the spatial overlap network reaches a maximum of 8%, but reduces again with more sampling. The false positive rate of the sequential trap sharing network remains between 2 and 3% (Fig. S3a).

### Sensitivity to the modelling framework

We found that our results were generally robust to our modelling assumptions. When using a different method to simulate our true network (uniform home ranges), the spatial overlap network remained more strongly correlated with the true network than the sequential trap sharing network when data were sparse, and comparably strongly correlated when more data were available (see Supplementary information; Fig. S1a). The spatial overlap network was also comparable in its power and accuracy to the sequential trap sharing network (Figs. S1b, S2b and S3c). However, we did not find that our results were robust to different implementations of spatial overlap networks. When using a different method to estimate home ranges and subsequent overlap between individual home ranges (MCPs), the spatial overlap network generally performed less well than the sequential trap sharing network (Fig. S4) except in terms of power to detect an effect, where it performed better than the sequential trap sharing network but less well than our own implementation (Fig. S2a). The false positive rate was comparable to that of the sequential trap sharing network (Fig. S3b).

### Performance of observed networks with varying effect sizes

Only the power of the observed networks changes as a result of varying effect sizes.

#### Power

As the effect size increases, there is a corresponding increase in the power of the sequential trap sharing network, above a mean of approximately 3 captures per individual. For example, at a mean of 4.3 captures per individual, there is a 2.3% chance of detecting a true positive if the effect size is half that found in our empirical data, 5.2% chance if the effect size is equivalent to that found in our empirical data, and a 24.5% chance if the effect size is twice that found in our empirical data. The spatial overlap network shows broadly the same pattern. At a mean of 4.3 captures per individuals, there is a 7.0% chance of detecting a true positive if the effect size is half that found in our empirical data, 11.8% chance if the effect size is equivalent to that found in our empirical data, and 28.8% chance if the effect size is twice that found in our empirical data. Below approximately 3 captures per individual, sequential trap sharing and spatial overlap networks have similar power, regardless of effect size.

### Changing trapping grid density

**Fig. 5:**
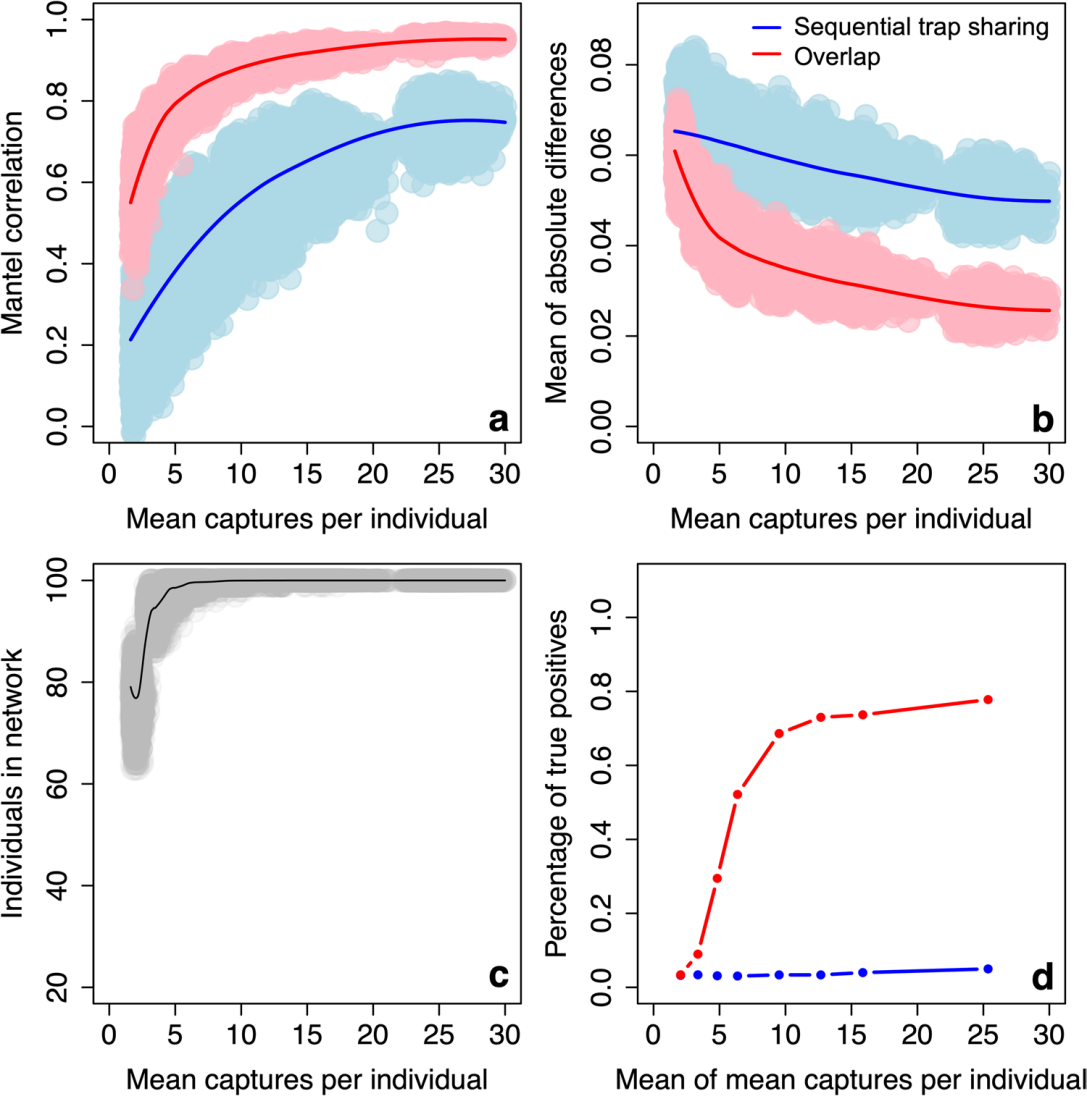
Performance of observed networks with varying numbers of captures per individual on a 19 × 19 trapping grid, as measured by **a** correlation, Mantel correlation between edge weights in observed and true networks; **b** accuracy, mean of absolute difference in edge weights between observed and true networks (lower values = more accurate networks); **c** number of individuals in observed networks; and **d** power, proportion of true positives. Individual data points are plotted and a LOESS smoother added to **a**–**c** to aid visual interpretation. Panel **c** refers to the data in the simulated observation dataset, which is identical for both methods

Observed networks change in all three metrics (correlation, accuracy and power) as a result of varying trapping grid density. Grid density also changes the number of individuals present in both observed networks when the number of captures per individual is low. For example, at 1–2 captures per individual, on a 19 × 19 grid, a mean of 77.2 individuals (out of a total population of 100 individuals) are present in the observed networks (compared to 31.3 on a 10 × 10 grid; see above). However, all individuals in the population are present in the observed networks (i.e. mean of 100.0 individuals) from a mean of approximately 10 captures per individual, regardless of grid density (Fig. 5c).

#### Correlation

A higher density grid leads to a weaker correlation between the sequential trap sharing network and the true network, for a given number of captures per individual (Fig. 5a). For example, at a mean of approximately 2 captures per individual, the Mantel correlation coefficient between the sequential trap sharing network and the true network is 0.4 on a 10 × 10 grid and 0.2 on a 19 × 19 grid. The sequential trap sharing network reaches the same maximum correlation coefficient of 0.8 on a 19 × 19 grid and on a 10 × 10 grid (see above). The correlation between the spatial overlap network and true network differs very little between the 10 × 10 and 19 × 19 grid (Fig. 5a). At a mean of approximately 2 captures per individual, the correlation coefficient between the spatial overlap network and the true network is 0.5 on a 10 × 10 grid and 0.6 on a 19 × 19 grid. The spatial overlap network also reaches the same maximum correlation coefficient of 1.0 on a 19 × 19 grid and on a 10 × 10 grid (see above).

#### Accuracy

A higher density grid leads to a slightly less accurate sequential trap sharing network for a given number of captures per individual (Fig. 5b). At a mean of approximately 2 captures per individual, the absolute difference in edge weights to the true network is a very similar 6.4 × 10^–2^ on a 10 × 10 grid and 6.5 × 10^–2^ on a 19 × 19 grid. However, the minimum absolute difference in edge weights for the sequential trap sharing network is slightly higher on a 19 × 19 grid (5.0 × 10^−2^) compared to a 10 × 10 grid (3.9 × 10^−2^; see above). A higher density grid has little effect on the accuracy of the spatial overlap network for a given number of captures per individual (Fig. 5b). For example, at a mean of approximately 2 captures per individual, the absolute difference in edge weights of the spatial overlap network to the true network is 6.1 × 10^−2^ on a 10 × 10 grid and 5.8 × 10^−2^ on a 19 × 19 grid. The minimum absolute difference in edge weights for the spatial overlap network is 2.3 × 10^−2^ on a 10 × 10 grid, and 2.6 × 10^−2^ on a 19 × 19 grid.

#### Power

The power of the sequential trap sharing networks changes very little with grid density (Fig. 5d). For example, at a mean of approximately 2 captures per individual, the chance of detecting a true positive is 3.1% on a 10 × 10 grid and 3.2% on a 19 × 19 grid. The power of the spatial overlap network also changes very little with grid density (Fig. 5d). At a mean of approximately 2 captures per individual, the chance of detecting a true positive is 3.0% on a 10 × 10 grid and 3.4% on a 19 × 19 grid.

## Discussion

In this study, we quantify the robustness of sequential trap sharing and spatial overlap networks to different sampling regimes. In doing so, we provide much needed guidance for informing the choice of sampling regime when designing studies to accurately quantify space sharing among individual animals. Using a large-scale empirical dataset for a population of field voles (*M. agrestis*), we also demonstrate the utility of modelling space-use behaviour on the basis that individuals have a core and a periphery to their home range. We then use these insights to develop a new method for generating shared space use networks based on estimating overlapping home ranges. We show that networks generated using the overlap method are generally more strongly correlated with the true shared space use network, are a more accurate representation of the true shared space use network and are more powerful to detect effects present in the true shared space use network relative to sequential trap sharing networks.

Our overlap method works particularly well when the mean number of captures per individual is low and provides the potential to generate meaningful networks even from sparse point-based observations of individuals. Compared to standard, more restrictive methods that rely only on sequential observations at a trap and sometimes impose a temporal threshold within which the observation of two individuals in the same place must occur, our method pools data among individuals to arrive at a more general estimate of home range profile. In doing so, our method accounts for imperfect and heterogeneous observations (as in e.g. Gimenez et al. 2019). Using these general profiles, we then calculate the extent of two individuals’ home range overlap, as a function of their observed centroids, to estimate their overlap in space. Our simulation results confirm that this approach results in more accurate and more representative networks than existing methods when data are sparse, mirroring similar findings from studies simulating social contacts using telemetry data (Gilbertson et al. 2021). However, our findings are sensitive to the choice of approach. We found that spatial overlap networks generally performed less well than sequential trap sharing networks when MCPs were used to estimate home range overlap, except in terms of power to detect a biological effect. MCPs are a non-data-hungry method for estimating home range, but suffer from some problems. They have the opposite problem at the boundary of two individuals’ home ranges, where two individuals can be assigned an edge weight of 0 when they were trapped at the same trap and, in reality, have some non-zero level of overlap. MCPs also assume a uniform density probability of occurrence across the home range. We acknowledge that MCPs are overly simplistic and encourage empiricists to find the method that best models the observed data from their animals when estimating their space use.

In our own implementation, we model differences in home range profile between large males and females based on our empirical data. However, classes of individuals that differ in their space use will vary between systems, and prior knowledge will be necessary to identify these classes, e.g. males and females, larger and smaller individuals or younger and older individuals (Wolton and Flowerdew 1985; Mikesic and Drickamer 1992; Dahle and Swenson 2003; Godsall et al. 2014). Given adequate classification and modelling, our results show that an increase in correlation and accuracy of the spatial overlap method using our own implementation translates to greater power at extracting biological effects present in the true shared space use network. In our case, we modelled differences in mean degree (sum of edge weights) between large males and females, but these outcomes should be generalisable to other hypotheses. As noted above, the spatial overlap network generated from MCPs was also more powerful than the sequential trap sharing network, but less powerful than our own implementation.

An important, and perhaps unexpected, finding is that denser grids of data collection traps can make sequential trap sharing networks less strongly correlated with the true shared space use network and less accurate (at least when the mean number of captures per individuals is below 30). This result makes sense when considering sampling stochasticity. The more traps are available, the less likely that two individuals, which overlap in space, will be trapped in *exactly* the same trap (unless the number of captures is very high and in no way limiting). Subsequently, constructing networks from occurrences at the same trap reduces the numerator of the edge weight calculation in Eq. 3 (the set of traps in which both individuals were detected) and increases the denominator (the set of traps in which at least one individual was detected). By contrast, we show that, in terms of correlation, accuracy and power, our spatial overlap method performs equally well, or better, on a denser trapping grid. This is because finer-scaled grids provide better estimations of individuals’ space use.

It is important to note that networks generated using the spatial overlap method do not always perform better than sequential trap sharing networks. We show here that the latter are more accurate and more powerful at detecting biological effects present in the true shared space use network when the effect size is small and the number of captures per individual is large (and the trap density is low). In other words, if many sequential trap sharing events are observed, then a network based on these alone deviates less from the true shared space use network and is more likely to detect a subtle biological effect present in the true network, relative to the process of pooling data and generating population-wide home range profiles. This finding aligns with the simulation results of Gilbertson et al. (2021), who found that—for telemetry data—spatial overlap networks also became overly dense and less sensitive at higher sampling rates (albeit, much higher sampling rates than could ever be achieved from trapping data). Thus, studies that use methods that produce substantially larger datasets than singular trapping does, such as RFID detections (e.g. Sabol et al. 2018), should model the sampling process to determine the most powerful approach for a given effect strength.

We further note that the performance of sequential trap sharing networks may also depend on the biological system or the ecological conditions it experiences (as in Perkins et al. 2009). Our study is based on empirical data on *M. agrestis* sampled during the breeding season. During this time, field voles maintain relatively fixed home ranges which can be estimated with some certainty (Myllymaki 1977; Niethammer and Krapp 1982). However, this is likely to be problematic if individuals are highly mobile, resulting in constantly shifting home ranges. We would expect a sequential trap sharing network, with some threshold in the time gap between detections, to be better suited to more dynamic systems. Our study population also inhabits a relatively homogenous landscape, in the form of grassy clear-cuts within a coniferous forest. As a result, individuals are not expected to vary a great deal in the size of their home range. Landscape features, such as hills, in a more heterogeneous landscape could result in more variability in home range size among individuals, making it difficult to quantify an ‘average’ home range. If sampling is sufficiently high (Noonan et al. 2019), individual differences in home range profile could be accounted for when using the overlap method. This could be done, for example, by fitting a random effect for individual within class-specific regressions. These individual home range profiles could vary in size (by changing the *a* and *b* parameters of the negative sigmoidal curve) and/or in shape (by replacing the negative sigmoidal curve with a different function or an explicit home range model, e.g. Fleming and Calabrese 2017). A number of approaches, beyond MCPs, also exist for modelling individual home ranges that could be employed (Winner et al. 2018). However, at higher sampling frequencies, a sequential trap sharing network could be better suited given the potential loss of performance from spatial overlap networks when individuals are detected very frequently (Gilbertson et al. 2021).

Shared space use networks are, and will continue to be, widely used to shed light on various biological processes. For example, individuals that share more space may be more likely to compete for resources. Many parasites and pathogens are also transmitted through the environment, and so, knowing who shares space with whom can tell us something about who is likely to transmit infection to whom (VanderWaal et al. 2013). It is also true that shared space use, or proximity, is a prerequisite for interaction (Farine 2015), but whether or not individuals that share space do indeed associate, or interact, and thus how far point-based observations can be used to draw meaningful inferences, will depend on the biology of the system. In some systems, a correlation between spatial overlap and direct contact has been described (Robert et al. 2012; Vander Wal et al. 2014), but behaviour in particular is important to consider, as some animals might actively avoid each other (Davis et al. 2014) whereas others might actively seek each other out (Raulo et al. 2021). For example, group-to-group social preferences (direct contact) in vulturine guineafowl (*Acryllium vulturinum*) multilevel societies are not correlated with home range overlap (Papageorgiou et al. 2019). It is therefore important to take care when making biological inferences from any network data.

One point we highlight in our study is that the process of network generation makes explicit assumptions about the biological processes being modelled. Using sequential trap sharing events, for example the presence of two individuals in the same location within a given pathogen transmission period (defined by the lifetime of the pathogen in the environment, or the time taken for a pathogen to develop into an infective stage), to produce a sequential trap sharing network produces networks aimed at estimating space-sharing events that actually took place and that may (or may not) have resulted in transmission. When observation data are sparse, these observed events are likely to represent only a small proportion of all events that took place, and thus, the power of the network to detect biological effects is low. This could explain why observed transmission networks are not always robust estimates of transmission processes (Wohlfiel et al. 2013). By contrast, more generally modelling the overlapping space use among individuals captures the relative probability of direct or indirect contact taking place among all the dyads in a population, which will include both observed and unobserved events. Our simulations confirm that defining networks in this way can produce networks that are more powerful at detecting biological effects, especially when observations are sparse (see also Gilbertson et al. 2021). We use pathogen transmission as an example to illustrate our point, but this should be generalisable to other questions.

Our method provides a novel opportunity to generate meaningful shared space use networks, and if appropriate, to make inferences from shared space use about social interactions, even from sparse point-based observations of individuals. It therefore unlocks the potential of these data, still the most common form of data available for many smaller or more cryptic species, to address a range of key questions in ecology and evolution.

## Supplementary Information

Below is the link to the electronic supplementary material.

Supplementary file1 (DOCX 5.18 MB)

## Data Availability

Data available from: https://datadryad.org/stash/dataset/doi:10.5061/dryad.bk537. The R code to run all of the simulations is available from: https://doi.org/10.17617/3.IASDTY**.**
