# Supplementary material for: A new method for characterising shared space use networks using animal trapping data
Source: Behav Ecol Sociobiol. 2022 Aug 26;76(9):127. doi: 10.1007/s00265-022-03222-5 (PMC9418289; doi:10.1007/s00265-022-03222-5)
Supplement: Supplementary file 1 — Supplementary file1 (DOCX 5.18 MB) [file 265_2022_3222_MOESM1_ESM.docx]

**SUPPLEMENTAL INFORMATION FOR:**

**A new method for characterising shared space use networks**

**using animal trapping data**

Klara M. Wanelik^1*^ & Damien R. Farine^2,3,4*^

^1^ Department of Evolution, Ecology and Behaviour, Institute of Infection, Veterinary and Ecological Sciences, University of Liverpool, Liverpool, UK.

^2^ Department of Evolutionary Biology and Environmental Studies, University of Zurich, Zurich, Switzerland.

^3^ Division of Ecology and Evolution, Research School of Biology, Australian National University, 46 Sullivans Creek Road, Canberra, ACT 2600, Australia.

^4^ Department of Collective Behaviour, Max Planck Institute of Animal Behavior, Konstanz, Germany.

^*^Corresponding authors

*Klara M. Wanelik*

Current address: Department of Biology, University of Oxford, Oxford, UK.

Email: [klara.wanelik@biology.ox.ac.uk](mailto:klara.wanelik@biology.ox.ac.uk)

*Damien R. Farine*

Email: [damien.farine@ieu.uzh.ch](mailto:damien.farine@ieu.uzh.ch)

Published in: **Behavioral Ecology and Sociobiology**

**
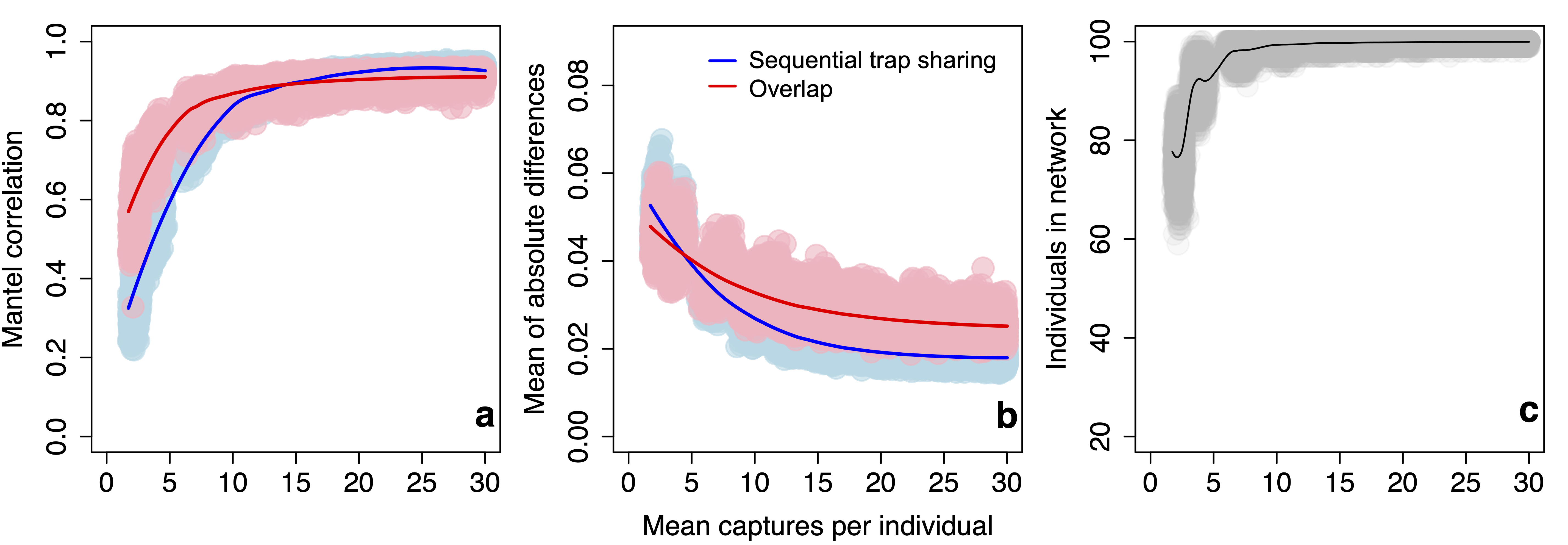
**

**Fig. S1** Performance of observed networks generated using our own implementation with varying numbers of captures per individual on a 10 × 10 trapping grid, as measured by (a) Correlation: Mantel correlation between edge weights in observed and true networks generated using uniform home ranges, (b) Accuracy: Mean of absolute differences in edge weights between observed and true networks generated using uniform home ranges (lower values = more accurate networks), and (c) Number of individuals in observed networks. ﻿LOESS smoother added to aid visual interpretation. Panel (c) refers to the data in the simulated observation dataset, which is identical for both methods.

**
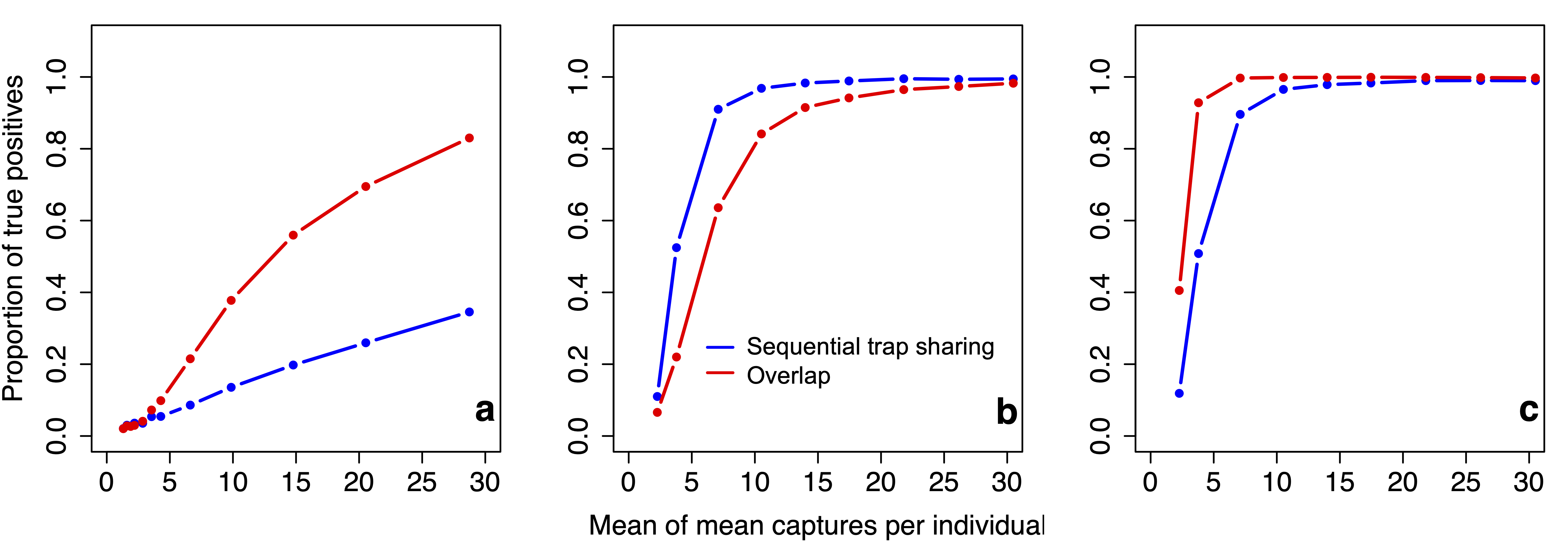
**

**Fig. S2** Performance of observed networks with varying numbers of captures per individual on a 10 × 10 trapping grid, as measured by the power of observed networks to detect a biological effect present in the true network. Proportion of true positives shown on *y*-axis, and mean of mean captures per individual shown on *x*-axis. Repeated for (a) observed networks generated using minimum convex polygons (MCPs) and true networks generated using our own implementation, (b) observed networks generated using our own implementation and true networks generated using uniform home ranges, (c) observed networks generated using MCPs and true networks generated using uniform home ranges.

**
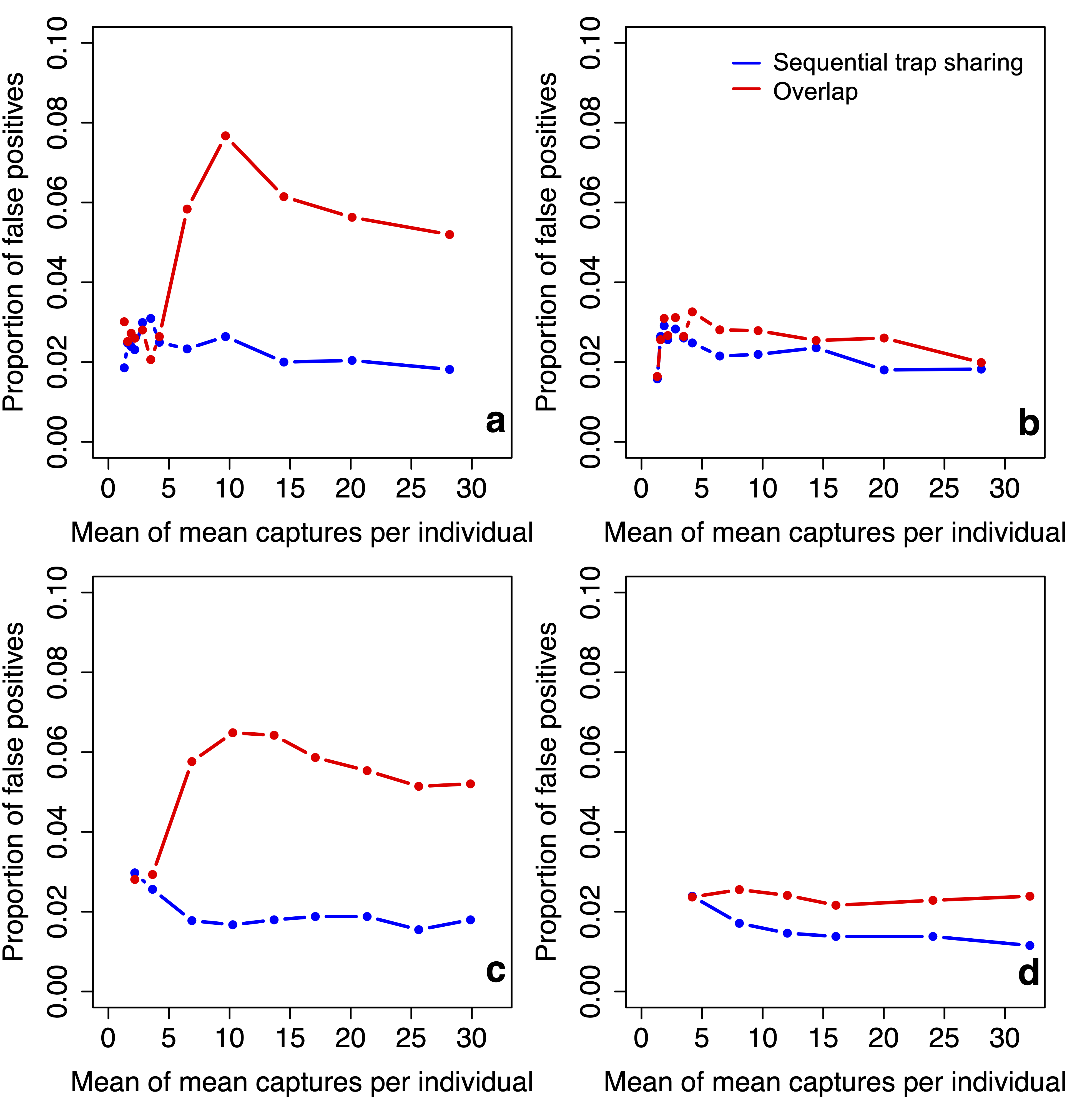
**

**Fig. S3** Performance of observed networks with varying numbers of captures per individual on a 10 × 10 trapping grid, as measured by the ability of observed networks to detect a biological effect present when it is not present in the true network. Proportion of false positives shown on *y*-axis, and mean of mean captures per individual shown on *x*-axis. Repeated for (a) observed networks and true networks both generated using our own implementation, (b) observed networks generated using minimum convex polygons (MCPs) and true networks generated using our own implementation, (c) observed networks generated using our own implementation and true networks generated using uniform home ranges, (d) observed networks generated using MCPs and true networks generated using uniform home ranges.

**
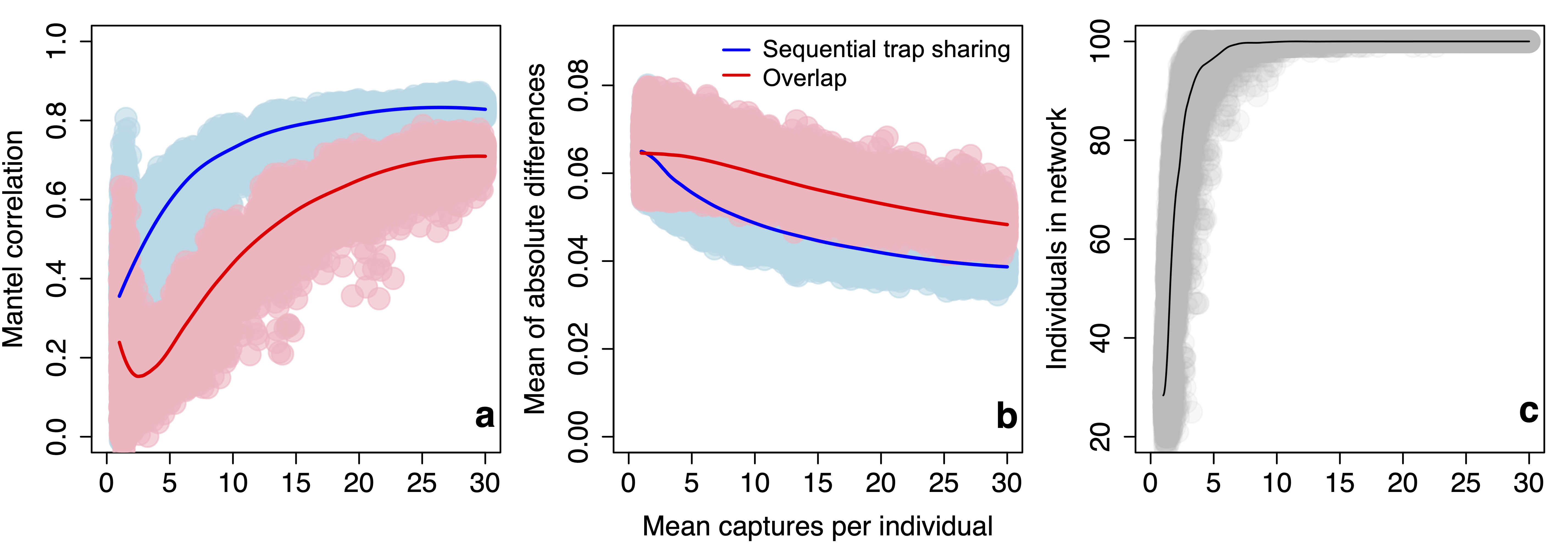
**

**Fig. S4** Performance of observed networks generated using minimum convex polygons (MCPs) with varying numbers of captures per individual on a 10 × 10 trapping grid, as measured by (a) Correlation: Mantel correlation between edge weights in observed and true networks generated using our own implementation, (b) Accuracy: Mean of absolute differences in edge weights between observed and true networks generated using our own implementation (lower values = more accurate networks), and (c) Number of individuals in observed networks. ﻿LOESS smoother added to aid visual interpretation. Panel (c) refers to the data in the simulated observation dataset, which is identical for both methods.

**
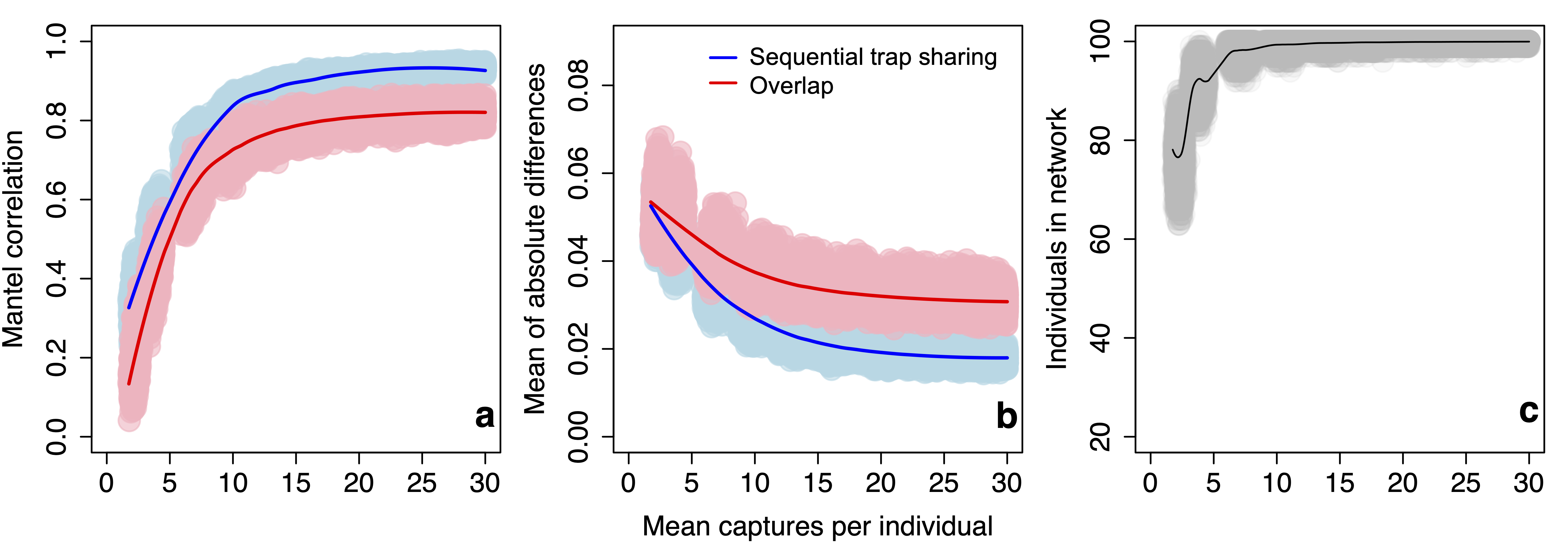
**

**Fig. S5** Performance of observed networks generated using minimum convex polygons (MCPs) with varying numbers of captures per individual on a 10 × 10 trapping grid, as measured by (a) Correlation: Mantel correlation between edge weights in observed and true networks generated using uniform home ranges, (b) Accuracy: Mean of absolute differences in edge weights between observed and true networks generated using uniform home ranges (lower values = more accurate networks), and (c) Number of individuals in observed networks. ﻿LOESS smoother added to aid visual interpretation. Panel (c) refers to the data in the simulated observation dataset, which is identical for both methods.
